# Bridging science and policy in tuberculosis treatment through innovations in precision medicine, drug development, and cohort research: a narrative review

**DOI:** 10.12771/emj.2025.00115

**Published:** 2025-04-02

**Authors:** Jinsoo Min, Bruno B. Andrade, Ju Sang Kim, Yoolwon Jeong

**Affiliations:** 1 Division of Pulmonary and Critical Care Medicine, Department of Internal Medicine, Seoul St. Mary’s Hospital, College of Medicine, The Catholic University of Korea, Seoul, Korea; 2 Laboratory of Clinical and Translational Research, Gonçalo Moniz Institute, Oswaldo Cruz Foundation, Salvador, Brazil; 3 The BRIGHT Lab, Multinational Organization Network Sponsoring Translational and Epidemiological Research Institute, Salvador, Brazil; 4 Division of Pulmonary and Critical Care Medicine, Department of Internal Medicine, Incheon St. Mary’s Hospital, College of Medicine, The Catholic University of Korea, Seoul, Korea; 5 Department of Preventive Medicine, Dankook University Hospital, Cheonan, Korea

**Keywords:** Drug-related side effects and adverse reactions, Iatrogenic disease, Precision medicine, Treatment outcome, Tuberculosis

## Abstract

Recent advancements in tuberculosis treatment research emphasize innovative strategies that enhance treatment efficacy, reduce adverse effects, and adhere to patient-centered care principles. As tuberculosis remains a significant global health challenge, integrating new and repurposed drugs presents promising avenues for more effective management, particularly against drug-resistant strains. Recently, the spectrum concept in tuberculosis infection and disease has emerged, underscoring the need for research aimed at developing treatment plans specific to each stage of the disease. The application of precision medicine to tailor treatments to individual patient profiles is crucial for addressing the diverse and complex nature of tuberculosis infections. Such personalized approaches are essential for optimizing therapeutic outcomes and improving patient adherence—both of which are vital for global tuberculosis eradication efforts. The role of tuberculosis cohort studies is also emphasized, as they provide critical data to support the development of these tailored treatment plans and deepen our understanding of disease progression and treatment response. To advance these innovations, a robust tuberculosis policy framework is required to foster the integration of research findings into practice, ensuring that treatment innovations are effectively translated into improved health outcomes worldwide.

## Introduction

### Background

Tuberculosis (TB) continues to pose an immense global health challenge. The World Health Organization (WHO) Global TB Report offers a sobering snapshot of the current epidemic: in 2023, an estimated 10.8 million people worldwide developed TB, corresponding to an incidence rate of approximately 134 cases per 100,000 population [1]. Although TB-related deaths declined modestly from 1.32 million in 2022 to 1.25 million in 2023, TB has re-emerged as the world’s leading infectious disease killer, surpassing coronavirus disease 2019. A particularly alarming finding is the diagnostic gap: only about 8.2 million of the estimated 10.8 million cases were detected, leaving roughly 2.7 million “missing” cases that fuel ongoing transmission. The situation is further complicated by the rising prevalence of drug-resistant TB; an estimated 400,000 cases of multidrug-resistant/rifampicin-resistant TB (MDR/RR-TB) were reported in 2023, yet only around 44% of these patients received appropriate treatment [1]. Parallel to these trends, current TB treatment strategies face several significant challenges. The discovery of effective medications such as streptomycin and para-aminosalicylic acid in 1944 marked the beginning of chemotherapeutic TB treatment. A pivotal advancement occurred in 1952 with the introduction of “triple therapy,” which combined streptomycin, para-aminosalicylic acid, and isoniazid; however, this regimen required up to 24 months of continuous treatment. Further progress emerged in the 1970s when combining isoniazid and rifampicin reduced treatment duration from 18 to 9 months, followed by the 1980s discovery that adding pyrazinamide allowed for cures in only 6 months. For the past 40 years, the global standard for TB treatment has been a 6-month regimen of isoniazid, rifampicin, pyrazinamide, and ethambutol, achieving success rates as high as 88%. Nevertheless, its prolonged duration, substantial pill burden, and side effects pose significant challenges for patient adherence [2], potentially leading to acquired drug resistance, treatment failure, and even death.

### Objectives

This review aims to critically assess recent advancements in TB treatment research and innovation, focusing on developing therapies that mitigate the limitations of current recommended treatments while aligning more closely with patient-centered care. By addressing persistent treatment challenges, we explore how recent research is paving the way for more effective and accessible interventions in the global fight against TB. This evaluation highlights the shift toward treatments that not only improve therapeutic outcomes but also enhance patient adherence and satisfaction.

## Ethics statement

As this study is a literature review, it did not require institutional review board approval or individual consent.

## Treatment strategy across the disease spectrum

**Fig. 1.**
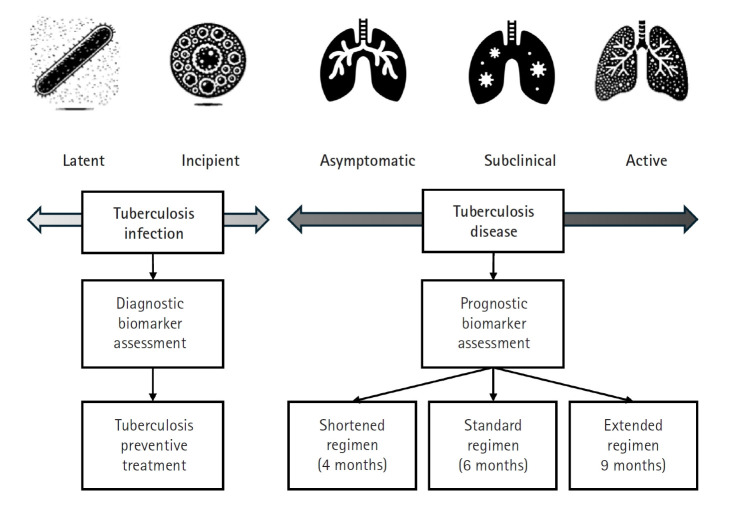
Continuum from tuberculosis infection to active tuberculosis disease. Starting with latent tuberculosis infection—where individuals harbor *Mycobacterium tuberculosis* without symptoms—the continuum progresses to incipient stages marked by metabolic and immunological changes that signal the early evolution of infection. It then enters an asymptomatic or subclinical phase, during which the disease is microbiologically active but exhibits minimal or no overt symptoms. Finally, the continuum culminates in active tuberculosis, characterized by symptomatic disease that necessitates comprehensive treatment. (Drawn by the authors.)

**Fig. 2.**
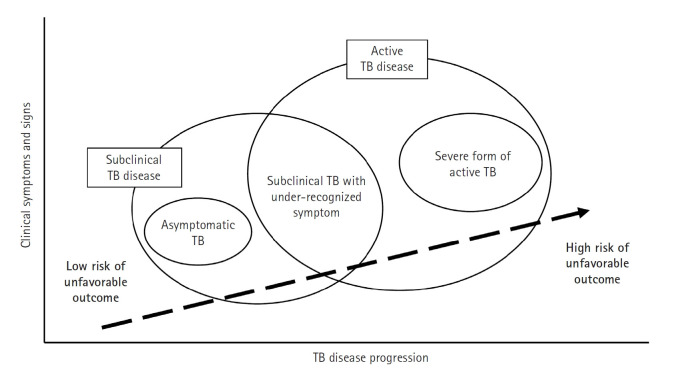
Progression of tuberculosis (TB) disease from a subclinical to an active state. This figure highlights the transition phase in which individuals may not show obvious symptoms despite ongoing microbiological activity (subclinical tuberculosis), progressing to active tuberculosis disease where symptoms become clinically evident and require immediate, intensive treatment. The upward movement in the diagram signifies a deterioration in the condition, emphasizing the critical need for early detection and intervention during the subclinical stage to prevent the full development of active tuberculosis and its complications. (Drawn by the authors.)

Recent research has significantly expanded our understanding of TB, moving beyond a simple classification of the infection as either latent or active. Instead, TB is now recognized as a disease spectrum encompassing several distinct stages, each with unique clinical and biological characteristics (Fig. 1) [3]. At one end of this spectrum is latent TB infection, a condition in which individuals harbor *Mycobacterium tuberculosis* without exhibiting any symptoms or other evidence of active TB. Although these individuals appear healthy, they remain at risk of progressing to more severe forms of the disease later in life. Moving along the continuum, the concept of incipient TB has emerged to describe an early stage of infection. In this phase, subtle changes—often detectable through sensitive immunological or radiographic methods—suggest that the infection is beginning to evolve toward a more active state, even though clinical symptoms are not yet apparent. Further along the spectrum lies subclinical TB [4], a stage in which the disease is microbiologically active and identifiable with advanced diagnostic tools, yet the affected individual may still exhibit minimal or no overt symptoms (Fig. 2).

Finally, at the most advanced end of the spectrum is active TB, where the infection manifests with clear clinical symptoms and necessitates prompt, often intensive, treatment. Comprehensive reviews emphasize that this continuum—or disease spectrum—provides a more accurate framework for understanding TB pathogenesis than the traditional binary model [5-7]. They highlight the benefits of adopting a stage-specific approach to treatment [5,6]. For example, individuals with latent or incipient TB might be managed with shorter, less aggressive regimens aimed at preventing disease progression, thereby reducing drug exposure and associated side effects. In contrast, patients with subclinical or active TB typically require the standard, comprehensive multidrug therapy to ensure both cure and transmission prevention. It is even possible that patients with limited subclinical TB could be treated with fewer drugs and shorter regimens. This nuanced perspective on TB not only improves diagnostic precision but also supports the development of tailored therapeutic strategies that address the specific needs of patients at different disease stages.

## Strategies to shorten tuberculosis treatment duration

**Fig. 3.**
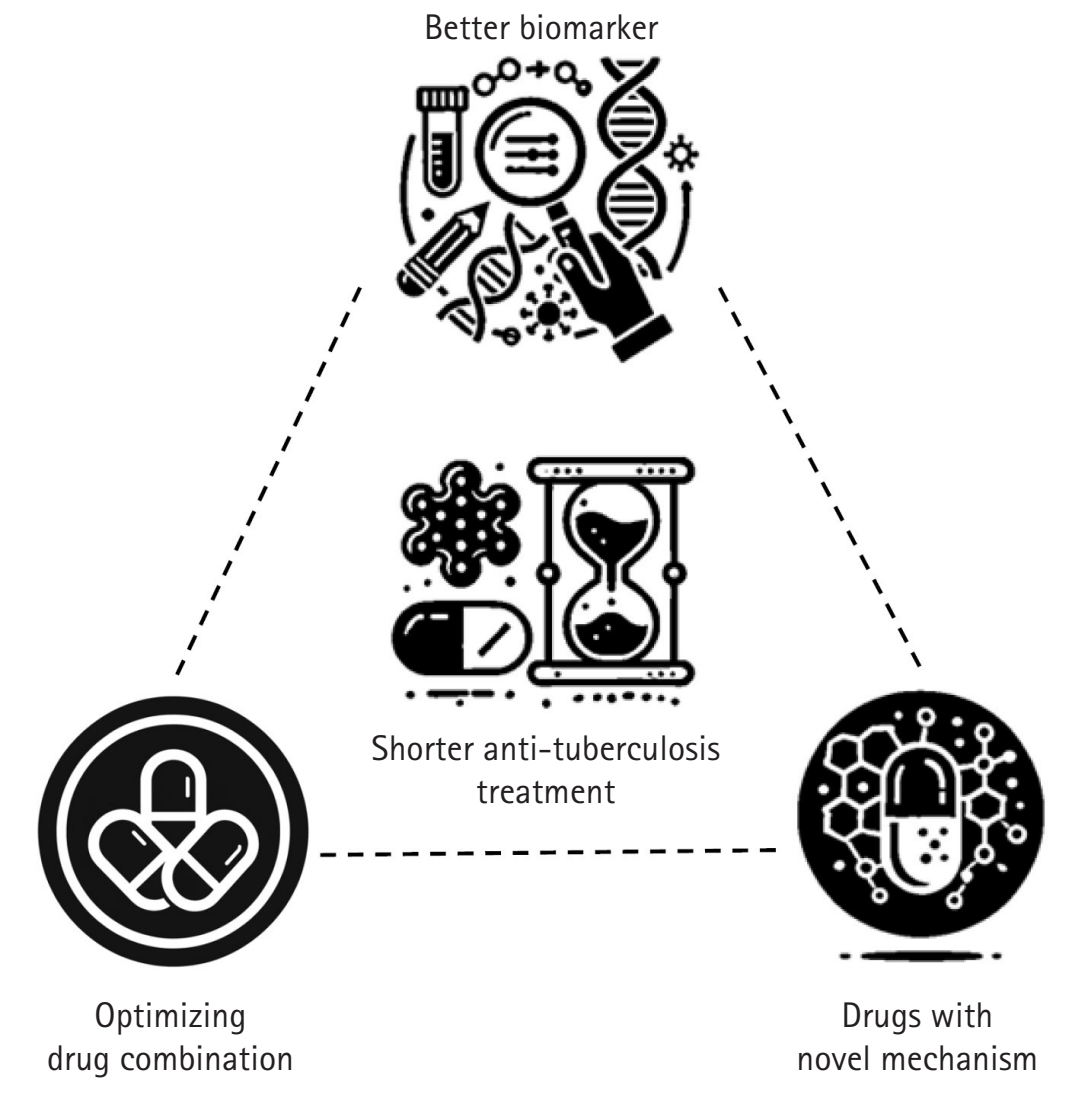
Strategies to shorten tuberculosis treatment. This diagram illustrates 3 key strategies aimed at reducing tuberculosis treatment duration while enhancing efficacy and patient outcomes. The “better biomarker” strategy focuses on developing and utilizing advanced biomarkers to tailor treatment duration and intensity to individual patient needs, thereby optimizing therapeutic outcomes. The “optimizing current drugs” strategy emphasizes enhancing the effectiveness of existing anti-tuberculosis medications through dose optimization and the exploration of synergistic drug combinations. Finally, the “novel mechanism drugs” strategy represents the exploration of new drugs and the repurposing of existing medications to target previously unexploited bacterial pathways, with the goal of overcoming drug resistance and further improving treatment efficacy. (Drawn by the authors.)

The foremost objective of TB treatment research is to develop rapid and effective therapies that can reduce the global spread of TB and lower TB-related mortality, ultimately contributing to disease eradication. To achieve this goal, researchers are focusing on shortening treatment duration while enhancing overall efficacy [8]. In practice, this involves developing short-course treatment regimens that improve patient adherence, minimize side effects, and ensure complete pathogen elimination. Three key strategies have been proposed to shorten TB treatment duration, each supported by extensive background research and clinical experience (Fig. 3).

The first strategy involves optimizing combinations of currently available anti-TB drugs [9]. By re-evaluating existing drug combinations, adjusting dosages and schedules, and exploring synergistic interactions, it is possible to enhance collective effectiveness, reduce treatment duration, and improve outcomes without increasing toxicity. The second strategy is to develop new drugs with novel mechanisms of action while repurposing existing drugs for TB treatment [10]. Most current anti-TB drugs target similar bacterial pathways, and over time, the bacteria have developed resistance to these mechanisms. Researchers are therefore focused on discovering compounds that act on previously untargeted pathways—such as unique components of the mycobacterial cell wall or other critical functions—while also repurposing drugs originally designed for other diseases that have proven effective against TB. These novel and repurposed drugs may offer greater potency, help overcome existing resistance, and contribute to a faster, more efficient treatment course. The third strategy is to identify and validate reliable prognostic biomarkers that can predict treatment outcomes [11].

A major challenge in TB treatment is the wide variation in patient responses; while some patients clear the infection rapidly, others experience relapse or treatment failure. By developing biomarkers—such as specific molecular signatures or immune profiles—that accurately reflect disease activity and predict prognosis, clinicians can tailor treatment duration and intensity to individual needs. This personalized approach not only minimizes unnecessary drug exposure and side effects but also ensures that high-risk patients receive the full benefit of extended therapy.

## Recent advances in drug discovery and development

The landscape of TB treatment has been enriched by the development and repurposing of several drugs, each with distinct mechanisms and clinical applications (Table 1). Bedaquiline disrupts the TB bacterium’s energy production by targeting adenosine diphosphate (ATP) synthase. Delamanid and pretomanid, both members of the nitroimidazoles class, exhibit antimycobacterial activity through a dual mechanism: they interfere with mycolic acid synthesis and induce respiratory poisoning [12].

Linezolid, an antibiotic that inhibits bacterial protein synthesis, was originally approved for drug-resistant Gram-positive infections and has proven effective against MDR/RR-TB.

Clofazimine, initially used for leprosy, has demonstrated potential in TB treatment by interfering with DNA replication. Research on high-dose rifampicin is currently underway to determine whether increasing the dosage from the traditional 10 mg/kg can increase efficacy and shorten treatment duration without raising adverse effects [13]. The global TB drug pipeline reveals numerous new therapeutic compounds at various stages of development [14]

Sudapyridine (WX-081) is a promising new anti-TB drug developed as an analog of bedaquiline [15]. It has demonstrated superior efficacy and safety in preclinical trials, showing excellent antimycobacterial activity and improved pharmacokinetic parameters with fewer side effects—such as reduced QTc prolongation—compared to its predecessor.

Currently, sudapyridine is undergoing Phase III clinical trials (NCT05824871). Telacebec (Q203) is a groundbreaking oral antibiotic that targets drug-resistant TB by selectively inhibiting the cytochrome bc1 complex of *M. tuberculosis*, which is crucial for the bacterium’s energy production. Initially developed by Qurient Co. Ltd., Telacebec showed promising results in a Phase 2a early bactericidal activity clinical trial (NCT03563599) that evaluated its safety, pharmacokinetics, and efficacy in adult, treatment-naïve, sputum smear-positive patients with drug-susceptible pulmonary TB [16]. The trial, completed in September 2019, confirmed Telacebec’s ability to significantly reduce the time to sputum positivity over the first 14 days of treatment, indicating potent bactericidal activity.

Delpazolid (LCB01-0371), a novel oxazolidinone developed by LegoChem BioSciences, has shown promise in early bactericidal activity trials, comparing favorably with the standard HRZE regimen and linezolid in reducing bacterial load in TB patients [17]. An ongoing Phase IIb clinical trial is assessing the safety, efficacy, tolerability, and pharmacokinetics of various doses of delpazolid in combination with bedaquiline, delamanid, and moxifloxacin over a 16-week treatment period in adult patients with newly diagnosed, smear-positive, drug-sensitive pulmonary TB (NCT04550832).

Another important strategy focuses on optimizing combination therapies to enhance efficacy and reduce treatment duration (Table 2). Researchers are re-assessing how current and novel drugs can be combined more effectively, exploring synergistic interactions that boost bactericidal activity while minimizing toxicity. The recommended regimen for drug-susceptible pulmonary and extrapulmonary TB, developed over 4 decades ago, consists of 6 months of isoniazid; rifampicin; pyrazinamide; ethambutol [18]. Numerous studies [19-21] that attempted to shorten treatment durations with fluoroquinolones failed to demonstrate non-inferiority until a successful 4-month regimen [22]—which included rifapentine, isoniazid, pyrazinamide, and moxifloxacin (4HPZM)—was developed. This regimen has received WHO endorsement for treating drug-susceptible TB in individuals aged 12 and older. Despite its success, challenges remain, including the need for ongoing support to ensure patient adherence and resistance testing for rifampicin and moxifloxacin, factors that may hinder its cost-effectiveness and broader implementation. Additionally, the availability of rifapentine remains restricted.

In Korea, concerns over the use of rifapentine complicate the adoption of the 4HPZM regimen. Rifapentine—a rifamycin antibiotic similar to rifampin but with a longer half-life that permits weekly dosing—was introduced in a pilot study in 2016 as a TB preventive treatment [23]. However, the study was prematurely terminated due to reports of anaphylaxis associated with rifapentine. Currently, rifapentine is not approved in Korea, and unresolved safety concerns make it difficult to adopt the WHO-recommended 4-month regimen. Further research and review are needed before this shorter regimen can be considered for use in Korea.

The Nix-TB trial demonstrated the efficacy of the BPaL regimen [24], which consists of bedaquiline, pretomanid, and linezolid, in curing patients with extensively drug-resistant TB or other difficult-to-treat DR-TB within 6 months, albeit in an uncontrolled study setting. Subsequently, the TB-PRACTECAL trial showed that adding moxifloxacin to BPaL (forming the BPaLM regimen) for 24 weeks was as effective as the WHO’s standard care for treating pulmonary MDR/RR-TB [25,26], while offering a better safety profile. In 2022, WHO endorsed BPaLM for 6 months as the new standard for MDR/RR-TB treatment in patients aged 14 and older who have not previously been treated with bedaquiline, pretomanid, or linezolid [27].

The BEAT India study, a prospective open-label, single-group cohort study, evaluated the effectiveness of a 24- to 36-week entirely oral regimen combining bedaquiline, delamanid, linezolid, and clofazimine (BDLC) in treating patients with pulmonary MDR-TB, including those resistant to fluoroquinolones or second-line injectables [28]. The study concluded that 91% of patients achieved a favorable outcome with minimal cardiotoxicity, although myelosuppression and peripheral neuropathy were common yet manageable side effects. WHO recently supported a 6-month regimen of bedaquiline, delamanid, linezolid, levofloxacin, and clofazimine (BDLLfxC) in the BEAT-TB study, which proved effective and safe for children, adolescents, and pregnant and breastfeeding women with MDR/RR-TB [29]. This trial employed an approach in which either levofloxacin or clofazimine was omitted from the regimen based on fluoroquinolone drug susceptibility testing results. The endTB study further explored combinations of these drugs, resulting in WHO guidance on 3 alternative 9-month, injectable-free regimens (BLMZ, BLLfxCZ, BDLLfxZ) that are contingent on fluoroquinolone susceptibility [30]. These regimens, tailored for both adults and children, demonstrate a continued commitment to refining TB treatment to enhance accessibility and effectiveness.

Alongside these approaches, the MDR-END regimen has been formulated as a 9-month all-oral combination of delamanid, levofloxacin, linezolid, and pyrazinamide, specifically designed for MDR/RR-TB [31]. Offering a robust therapeutic option for patients with drug-resistant TB, this regimen is now recommended as one of the first-line therapies for MDR/RR-TB according to the recently updated Korean TB treatment guidelines. Additionally, a non-interventional, prospective observational registry is currently underway in Korea to assess the real-world efficacy and safety of the MDR-END regimen.

The TRUNCATE trial demonstrated that shorter treatment regimens for rifampicin-susceptible TB are feasible when a risk-stratified approach is applied [32]. In this trial, patients with mild TB who had no risk factors for treatment failure or recurrence were successfully treated with an 8-week regimen. This regimen, which included both first-line and second-line drugs such as bedaquiline, linezolid, isoniazid, pyrazinamide, and ethambutol, proved to be as effective as the standard longer treatments. This finding suggests that the majority of TB patients might be cured in significantly less time than the conventional 24 weeks if similar approaches are implemented. Identifying the smaller group of patients who require longer treatment due to more complex cases remains a focus of current research.

## Host-directed therapies

Host-directed therapies (HDTs) can significantly optimize TB treatment by addressing several critical challenges encountered with current regimens. By modulating the host’s immune response to better control and eliminate *M. tuberculosis*, HDTs have the potential to shorten treatment duration—a key advantage for patients with drug-resistant TB strains who often require prolonged therapy [33]. This reduction in treatment time minimizes exposure to toxic drugs and decreases the likelihood of further resistance development. Moreover, HDTs can enhance the efficacy of existing TB medications by boosting the host’s immune capacity to fight the infection more effectively [34]. This improvement is particularly important for patients with compromised immune systems, such as those co-infected with human immunodeficiency virus (HIV), where conventional TB treatments may be less effective. By strengthening the immune response, HDTs help these patients better manage and overcome TB. Additionally, HDTs focus on reducing inflammation and preventing tissue damage caused by both the disease and its treatment. Excessive inflammation, a hallmark of severe TB, can lead to extensive lung damage that worsens patient outcomes and hinders recovery. By controlling inflammatory responses, HDTs not only protect lung tissue and preserve lung function but also improve overall treatment outcomes. This approach holds promise as a potential option for managing post-TB lung disease [35], offering a way to mitigate long-term respiratory complications and enhance recovery.

## Development of biomarkers

**Fig. 4.**
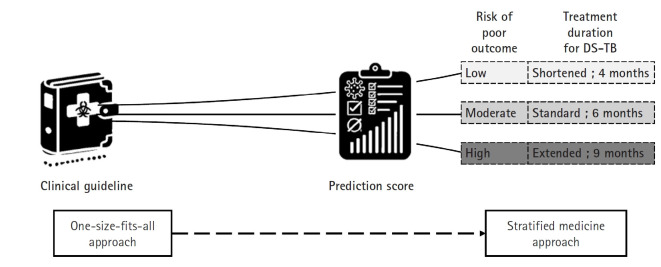
Treatment approaches in tuberculosis management. This diagram contrasts 2 distinct approaches to tuberculosis treatment. The traditional one-size-fits-all approach involves administering the same treatment regimen to all patients regardless of individual differences. In contrast, the stratified medicine approach uses a clinical scoring system to determine treatment duration based on a patient’s risk level—low, moderate, or high—thereby tailoring therapy to individual needs. This strategy aims to optimize outcomes by adjusting treatment length according to disease severity and prognosis, ultimately enhancing both efficacy and patient adherence. DS-TB, drug-susceptible tuberculosis. (Drawn by the authors.).

The current treatment guidelines for drug-susceptible TB advocate a uniform 6 month short-course regimen for all patients—a “one size fits all” approach (Fig. 4). However, clinical observations reveal that TB is a heterogeneous disease; some patients display a favorable prognosis and might safely undergo treatment shortening to as little as 4 months, while others, such as those who remain culture-positive at 2 months or who exhibit cavitary lesions on initial chest X rays, face a higher risk of relapse and may benefit from extending therapy to 9 months [36]. Unfortunately, the scientific evidence supporting these adjustments is still limited.

In recent years, interest in measuring the host response to TB has grown. Biomarker tests that detect blood RNA signatures and other analytes have demonstrated the capacity to distinguish between different TB disease states [3]. The development of robust prognostic biomarkers would enable a biomarker-guided therapy strategy, allowing clinicians to predict a patient’s likely treatment outcome from the outset. For example, prediction scores that combine clinical information—such as body mass index and time to sputum culture positivity—with markers of host response have shown promise, particularly since a high baseline mycobacterial load is an important predictor of relapse. One innovative example is the Xpert MTB Host Response (MTB-HR) prototype (Cepheid). This new fingerstick blood test generates a “TB score” based on the mRNA expression of 3 genes, providing a rapid, non-sputum-based, point-of-care test. In trials conducted across multiple countries, the MTB-HR prototype demonstrated high sensitivity and specificity in distinguishing TB from other respiratory diseases and showed potential for monitoring TB treatment response [37-39].

With validated biomarkers, patients expected to have a good prognosis could receive a shortened regimen (for example, 4 months), whereas those predicted to have a poorer prognosis could be assigned an extended regimen (up to 9 months) [40]. This “stratified medicine” approach would not only individualize therapy—optimizing treatment efficacy and minimizing unnecessary drug exposure and side effects—but also improve patient adherence. Furthermore, integrating these biomarkers into TB clinical research can enhance trial design by enabling more precise patient stratification. This refinement would lead to better interpretation of treatment responses, more efficient clinical trials, and ultimately, more personalized and effective TB treatment strategies on a global scale. An accessible, point-of-care relapse prediction score would accelerate the development and implementation of shorter, individualized TB treatment regimens.

## Advancing TB treatment research through cohort studies: a global perspective

TB treatment research is undergoing a transformative shift, driven by an expanding arsenal of new and repurposed compounds and increasingly sophisticated clinical trial methodologies [41]. While randomized controlled trials (RCTs) remain the gold standard for establishing treatment efficacy and informing public health guidelines, cohort studies provide an indispensable complementary approach. By capturing real-world, longitudinal data from diverse populations, cohort studies illuminate the complexities of TB pathogenesis, treatment responses, and long-term patient outcomes that controlled trials often cannot fully capture [42,43]. Cohort studies offer a critical advantage by investigating TB in naturalistic settings, encompassing a broad spectrum of patients who might otherwise be excluded from RCTs due to stringent eligibility criteria. This inclusivity ensures that TB treatment strategies reflect the heterogeneous nature of affected populations, including pregnant and lactating women, children, the elderly [44], and individuals with comorbid conditions such as diabetes [45] and HIV/acquired immunodeficiency syndrome.

Additionally, cohort studies facilitate the evaluation of treatment effectiveness and safety across varied demographic and geographic settings, generating data that more accurately reflect routine clinical practice. Beyond clinical outcomes, cohort studies serve as a powerful tool for deciphering TB disease progression, tracking the entire spectrum from latent TB infection and asymptomatic TB to active disease and post-treatment relapse [46]. The ability to monitor patients over extended periods enables researchers to assess host–pathogen interactions, immune responses, and environmental or genetic factors that influence TB trajectory [47]. This longitudinal framework is instrumental in identifying biomarkers that can predict treatment response, disease recurrence, and even progression from infection to active disease—key areas where existing diagnostic and prognostic tools remain insufficient. One of the most pressing challenges in TB research is the need for reliable biomarkers that can expedite drug development and optimize treatment regimens [48]. Current clinical trials rely on treatment success and relapse rates as primary endpoints, requiring prolonged follow-up periods that significantly delay the translation of new therapies into practice. The 2-month sputum culture conversion, commonly used as an early marker of treatment response, has limited predictive value, necessitating large sample sizes and extended study durations [49]. This inefficiency underscores the urgency of developing more precise host-derived and pathogen-based biomarkers to serve as early surrogates for treatment outcomes [50].

Cohort studies provide an ideal framework for identifying and validating such biomarkers. By systematically collecting longitudinal data on clinical, microbiological, immunological, and molecular markers, cohort studies enable researchers to correlate specific biomarkers with disease progression and treatment response [51]. This approach facilitates the development of personalized TB treatment strategies, allowing for tailored regimens that minimize toxicity, reduce treatment duration, and improve adherence. The integration of advanced omics technologies, including transcriptomics, proteomics, and metabolomics, into cohort research holds immense potential for uncovering novel predictors of treatment success and relapse, paving the way for adaptive clinical trial designs that can accelerate drug evaluation and enhance patient stratification [52]. The push toward precision medicine in TB hinges on understanding individual variations in treatment response and disease susceptibility [53]. Unlike conventional clinical trials that focus on population-level outcomes, cohort studies allow for deep phenotyping of TB patients by integrating host genetics, microbiological characteristics, immune responses, and environmental exposures into predictive models. This approach has the potential to revolutionize TB treatment by shifting from one-size-fits-all regimens to more targeted, individualized therapies [54].

Furthermore, cohort studies provide a dynamic platform for evaluating new therapeutic strategies outside the constraints of traditional RCTs. The use of real-world evidence derived from these studies is increasingly recognized as a critical component in regulatory decision-making, bridging the gap between experimental treatments and their practical implementation in endemic settings [55]. Given the diversity of TB manifestations across different populations, leveraging cohort study data ensures that treatment recommendations are both context-specific and globally applicable [56].

The Regional Prospective Observational Research in Tuberculosis (RePORT) International consortium exemplifies the power of global collaboration in cohort-based TB research. Operating across Brazil, India, South Africa, China, Indonesia, the Philippines, Uganda, and the Republic of Korea, RePORT International fosters the development of harmonized, prospective cohort studies that generate high-quality, standardized data for cross-regional analyses. By unifying research efforts across diverse epidemiological landscapes, the consortium facilitates comparative studies on TB transmission, treatment response, and biomarker discovery, thereby amplifying the impact of cohort research on a global scale.

With the recent integration of RePORT Korea, in collaboration with the Korea National Institute of Health and the National Institute of Infectious Diseases, the RePORT framework continues to expand its global reach. This integration strengthens data-sharing initiatives, harmonized protocols, and collaborative research networks, enabling Korean researchers to both contribute to and benefit from international TB research efforts. Moreover, RePORT Korea’s engagement with the Cohort Study of Pulmonary Tuberculosis (COSMOTB) [57]—a prospective observational study encompassing nearly 3,000 patients across 20 hospitals—underscores the growing momentum of global TB cohort studies in advancing treatment research and biomarker discovery.

By aligning with RePORT International’s commitment to standardized methodologies and open data-sharing, RePORT Korea is poised to make significant contributions to biomarker-driven TB research, potentially accelerating the development of novel diagnostic and prognostic tools. This level of global integration is necessary for overcoming the persistent challenges in TB treatment research, driving innovation in drug development, clinical trial efficiency, and personalized medicine approaches.

The next frontier in TB treatment research lies at the intersection of cohort studies, precision medicine, and biomarker-driven clinical trials. While randomized trials remain fundamental for evaluating treatment efficacy, cohort studies provide the essential context, diversity, and longitudinal data required to refine therapeutic strategies, predict treatment success, and improve patient outcomes. By leveraging global research consortia such as RePORT International and initiatives like COSMOTB, the TB research community can accelerate the discovery of biomarkers, optimize treatment regimens, and ultimately redefine the paradigm of TB management. These efforts will enhance clinical decision-making, improve patient care, and propel TB eradication efforts forward, bridging the persistent gaps in our understanding of this complex disease.

## Connecting TB treatment research to TB policy

To effectively combat TB, it is crucial to connect TB treatment research with policy-making. This connection enables the formulation of strategies that directly address the evolving challenge of drug resistance and improve treatment outcomes globally. Successful TB research hinges on effective collaboration among various stakeholders—including government bodies, private industries, and academic institutions—which is vital for pooling resources, sharing knowledge, and driving innovation in support of large-scale, complex research projects.

Furthermore, adherence to a global strategy that promotes sustained investment in TB research is indispensable. Such a strategy fosters innovation, ensures data transparency, and improves access to new discoveries, thereby facilitating the creation of evidence-based TB policies. These policies are essential because they rely on scientific data to inform decisions that enhance the effectiveness, efficiency, and reach of TB treatment programs.

Implementing clear and robust regulatory and ethical frameworks ensures that research activities align with the needs and priorities of those most at risk. This alignment helps build trust, enhances the recruitment and retention of study participants, and ensures that research outcomes are both relevant and accepted. Active community engagement is also pivotal, as it ensures that TB treatment research addresses the real-world conditions of affected populations, making the research not only ethical but also practical and applicable.

By integrating these elements, TB research can significantly enhance the potential for developing effective new treatments, thereby improving patient outcomes and contributing to the global eradication of TB. This comprehensive approach ensures that advancements in TB treatment are scientifically robust and appropriately tailored to meet the diverse needs of populations affected by this devastating disease, all underpinned by evidence-based policymaking that ensures the sustainability and relevance of TB control efforts.

Korea, despite being a high-income country, continues to face significant challenges with TB [58]. In response, the third national strategic plan for TB control was introduced in 2023, focusing on the full spectrum of TB management—including prevention, diagnosis, and treatment. This plan emphasizes the development of innovative therapeutic agents and treatment strategies using cutting-edge technologies such as artificial intelligence and omics data. Additionally, it supports creating an optimized evaluation system for preclinical and nonclinical stages, as well as a nationally led infrastructure for standardizing and sharing research resources. These efforts are intended to bolster scientific advancements in TB treatment and ensure effective, patient-centered therapies grounded in robust scientific evidence in Korea.

## Challenges in TB treatment development and deployment

To effectively address the multifaceted challenges of TB treatment development, a holistic approach is required that spans from the laboratory to local communities. First, the process of drug development for TB faces significant hurdles. High attrition rates in TB drug development often stem from difficulties in translating preclinical results into safe, effective, and regulatory-approved therapies. Financial limitations further restrict the advancement of promising treatments due to the high costs associated with extensive clinical testing. Additionally, stringent regulatory requirements frequently cause significant delays. Strategic partnerships and policy reforms are essential to bridge these gaps and reduce the time from discovery to clinical application.

Second, the implementation of TB treatment guidelines varies significantly across different healthcare environments. These guidelines, primarily based on data from controlled clinical trials, may not always align with the diverse realities of various regions—especially in areas with unique epidemiological profiles, healthcare infrastructures, and cultural practices. To ensure global efficacy, it is crucial to assess the adaptability of these guidelines to local conditions. This might involve customizing treatment protocols to better suit regional healthcare settings, thereby making the guidelines more relevant and effective worldwide.

Lastly, the real-world deployment of new TB treatments is often hindered by several obstacles, particularly in regions with limited resources where TB is most prevalent. Key challenges include limited access to the latest medications—especially in low-resource settings where the TB burden is highest—and healthcare infrastructures that may lack the capacity to implement new protocols effectively. Additionally, socioeconomic barriers such as poverty, lack of education, and inadequate healthcare coverage can restrict patient access to treatment and follow-up care, severely affecting adherence rates and overall treatment success. Enhanced efforts to improve medication accessibility, strengthen healthcare capacities, and implement supportive policies are required to overcome these barriers, ensuring that advancements in TB treatment are effectively realized and benefit those in need.

By addressing these interconnected aspects, from drug development and guideline adaptability to practical rollout challenges, this comprehensive strategy aims to optimize TB treatment at all levels and significantly advance global TB eradication efforts.

## Conclusion

Integrating cutting-edge TB treatment research into TB policies is crucial for effectively addressing the global TB epidemic. Innovations in drug development and strategic treatment optimization are necessary to tackle the challenges of drug resistance and improve treatment outcomes. Implementing a comprehensive approach that improves the accessibility and effectiveness of TB treatments requires strong collaboration among governments, private sectors, and academic institutions, along with a steadfast commitment to evidence-based policymaking.

## Figures and Tables

**Table 1.** Overview of current and developmental tuberculosis treatment drugs and their mechanisms of action

Types	Examples	Class	Mechanism of action
New drugs	Bedaquiline	Diarylquinolines	Inhibits ATP synthesis by targeting mycobacterial ATP synthase
	Delamanid, pretomanid	Nitroimidazoles	Inhibits mycolic acid synthesis
	Telacebec	Imidazopyridines	Inhibits cytochrome bc1 complex, disrupting energy production
Repurposed drugs	Linezolid, depazolid	Oxazolidinones	Inhibits protein synthesis, binds to the 23S RNA peptidyl transferase center of the prokaryotic ribosomal 50S subunit
Revived drugs	Clofazimine	Riminophenazines	Interferes with DNA and cellular functions, anti-inflammatory effects
Optimized drugs	Rifampicin, rifapentine	Rifamycines	Inhibits DNA-dependent RNA polymerase, blocking RNA transcription

ATP, adenosine diphosphate.

**Table 2.** Currently available treatment regimens for drug-susceptible tuberculosis and multidrug-resistant/rifampicin-resistant tuberculosis

Target	Treatment regimen	Duration (mo)	Evidence
DS-TB	INH, RIF, PZA, EMB	6	BMRC [18]
	RFP, INH, PZA, Mfx	4	Study 31/A5349 [22]
Fluoroquinolone-susceptible MDR/RR-TB	Bdq, Pa, Lzd, Mfx	6	TB-PRACTECAL trial [25,26]
	Bdq, Dlm, Lzd, Lfx, Cfz	6	BEAT-TB study [29]
	Dlm, Lfx, Lzd, Pza	9	MDR-END study [31]
	Bdq, Lzd, Mfx, Pza	9	EndTB study [30]
	Bdq, Cfz, Lzd, Lfx, Pza	9	EndTB study [30]
	Bdq, Dlm, Lzd, Lfx, Pza	9	EndTB study [30]
Fluoroquinolone-resistant MDR/RR-TB	Bdq, Pa, Lzd	6	Nix-TB trial [24]
	Bdq, Pa, Lzd, Cfz	6	BEAT-India study [28]

DS-TB, drug-susceptible tuberculosis; MDR/RR-TB, multidrug-resistant/rifampicin-resistant tuberculosis; INH, isoniazid; RIF, rifampicin; PZA, pyrazinamide; EMB, ethambutol; RFP, rifapentine; Mfx, moxifloxacin; Bdq, bedaquiline; Pa, pretomanid; Lzd, linezolid; Dlm, delamanid; Cfz, clofazimine.
